# The role of a cryocompression device following total knee arthroplasty to assist in recovery: a randomised controlled trial

**DOI:** 10.1007/s00167-023-07455-3

**Published:** 2023-07-18

**Authors:** Mira Marinova, Abayasankar Sundaram, Katie Holtham, Jay R. Ebert, David Wysocki, Daniel Meyerkort, Ross Radic

**Affiliations:** 1 Perth Orthopaedic and Sports Medicine Research Institute, 31 Outram St, West Perth, WA 6005 Australia; 2 Perth Orthopaedic and Sports Medicine Centre, 31 Outram St, West Perth, WA 6005 Australia; 3 School of Human Sciences (Exercise and Sport Science), University of Western Australia, Crawley, WA Australia; 4 Division of Surgery, School of Medicine, University of Western Australia, Crawley, WA Australia; 5 Department of Orthopaedics, Royal Perth Hospital, Perth, WA Australia; 6 Sports Physiotherapist, Beatty Park Physiotherapy, North Perth, Australia

**Keywords:** Total knee arthroplasty, Cryocompression, Rehabilitation, Game Ready™, Patient-reported outcome measures, Pain, Clinical outcomes, Range of motion, Patient satisfaction

## Abstract

**Purpose:**

The study sought to investigate the effectiveness of a cryocompression Game Ready™ (GR) versus usual care protocol (UC) on early post-operative recovery following total knee arthroplasty.

**Methods:**

This study prospectively randomised 72 total knee arthroplasties to a 2-week (from day 0) intervention of GR treatment (*n* = 36, 63.9% females) or UC of ice with static compression (*n* = 36, 45.7% females). Knee flexion and extension range of motion (ROM), a visual analogue pain scale and limb circumference were documented at day 1, 2 and 14, as well as 6 weeks post-surgery. Medication usage and length of hospital stay were documented. Patient-reported outcome measures (PROMs) included the Knee Injury and Osteoarthritis Outcome Score and a Patient Satisfaction Questionnaire. Statistical analysis using linear mixed modelling and analysis of variance table with Satterthwaite's method were used along with two-tailed t-tests.

**Results:**

There were no significant group-by-time interactions regarding any of the outcomes. The GR group had 19% lost to follow-up at 2 weeks, while the UC group had 8%. The GR group demonstrated significantly better knee extension ROM at day 1 (*p* = 0.048) and day 14 (*p* = 0.007) compared with the UC group. There were no group differences (n.s.) observed in pain, flexion ROM, limb circumference, opioid use or PROMs. Overall, higher pain levels resulted in increased opioid intake (*p* = 0.002), older patients used significantly less opioids (*p* < 0.001) and males reported significantly less pain than females (*p* = 0.048). No adverse effects were observed due to either protocol.

**Conclusion:**

Despite patients gaining significantly more knee extension during the initial two-week intervention period when using GR compared to UC, this effect was likely due to chance. No further significant differences were observed between the groups during or after cession of the intervention.

**Level of evidence:**

Level 2.

## Introduction

Pain and inflammation are expected during the recovery period immediately following total knee arthroplasty (TKA), given the extensive soft tissue damage and blood loss caused [1]. Although acute inflammation promotes tissue healing, it is postulated that secondary injury caused by hypoxia and the release of enzymes can result in tissue damage and cell death [17, 19]. Excessive inflammation may lead to undesirable post-operative complications such as arthrofibrosis [29]. By mobilising patients and encouraging range of motion (ROM) exercises early, hospital length of stay (LOS) and the risk of post-operative complications can be reduced [15, 33]. Furthermore, a heightened post-operative pain experience can lead to central and peripheral sensitisation resulting in prolonged pain issues and delayed recovery [10]. Adequate analgesia is important to enhance rehabilitation, [9] and the use of opioid analgesia should be minimised where possible, given the potential side effects [36].

Cryocompression therapy is a non-invasive and non-pharmacological modality used in managing acute inflammation and pain, demonstrating benefits in the post-operative setting promoting vasoconstriction, reducing blood flow and inflammation [27]. Applying cold therapy has shown to decrease post-operative pain and opioid consumption following TKA [5, 28]. The cooling of tissue slows neuronal conduction velocity and transmission which in turn decreases muscle spasms, decreases metabolic demand and safely relieves pain without the use of narcotics [11]. Compression therapy has also shown its own benefits particularly in swelling reduction along with reduction of deep vein thrombosis (DVT) in the lower limbs following TKA [16]. Combining compression to cryotherapy may offer added benefits than either treatment in isolation [14, 18].

While elastic wraps and icepacks are commonly used modalities in the post-TKA setting, cryocompression therapy is not routinely implemented post-operatively. The Game Ready GRPro® 2.1 (GR) system offers a novel, easy to administer method of administering constant cryotherapy at a consistent temperature with intermittent pneumatic compression. The limited literature evaluating the GR system demonstrates it is a safe, user friendly device with improved outcomes and patient satisfaction [21, 23] but reflects ongoing inconsistency and lack of evidence. Given the increasing popularity of the GR system, this study sought to investigate the benefit in using a GR protocol following TKA to assist in post-operative rehabilitation. The hypothesis is that the use of GR will reduce pain and opioid consumption along with improving clinical outcomes after TKA.

## Materials and methods

A single centre, prospective randomised controlled trial (RCT) was undertaken to evaluate the benefit of a post-operative cryocompression protocol using the GRPro^®^2.1 cryocompression system (formerly CoolSystems Inc., Concord, CA, USA; now Avanos Medical Inc., Alpharetta, GA, USA), versus UC in the early recovery period following TKA. Patients > 18 years of age, presenting with end-stage knee osteoarthritis and scheduled to undergo primary TKA with one of three experienced knee surgeons, were invited to participate in the trial. Patients were excluded if they presented with a body mass index (BMI) > 40, had a history of cognitive impairment, had a history of prior infection, neurovascular compromise or a strong desire to follow a particular post-operative icing protocol which did not align with the study protocol. Patients were randomised into one of the two groups (GR or UC) as outlined below, with randomisation undertaken by a research coordinator using a ‘random number generator’ (1 = GR, 2 = UC). All participants provided informed consent prior to participating in the study.

### Surgery

TKA was performed with the Rosa® Knee System (Zimmer Biomet, Warsaw, IN, USA) using the Persona® Knee implant. Surgery was performed under spinal anaesthesia along with an adductor canal block and local anaesthetic infiltration. Antibiotics (2 g cefazolin) and tranexamic acid (1 g) were given at the time of induction and continued for 24 h post-operatively. Both groups followed the same post-operative multimodal analgesia regime, with patients receiving standardised post-operative analgesia which included paracetamol (1 g 6 hourly), non-steroidal anti-inflammatories (200 mg celecoxib 12 hourly), if tolerated, and slow-release (SR) opioid-based medication with top-up opioid analgesia as needed. Patients primarily received 50–100 mg of tapentadol SR along with immediate-release (IR) tapentadol. Where this was not tolerated, it was substituted for oxycodone with naloxone SR and oxycodone IR. Dressings were applied after wound closure which remained intact for 2 weeks following surgery and compressive crepe bandaging was applied in theatre, which was removed the following day. Both groups were able to fully weight bear on day one and followed the same standardised rehabilitation protocol guided by the in-patient physiotherapy team, along with routine day one post-operative x-rays, bloods and thromboembolic prophylaxis.

### Usual care (UC) and game ready (GR) treatment protocols

**Fig. 1:**
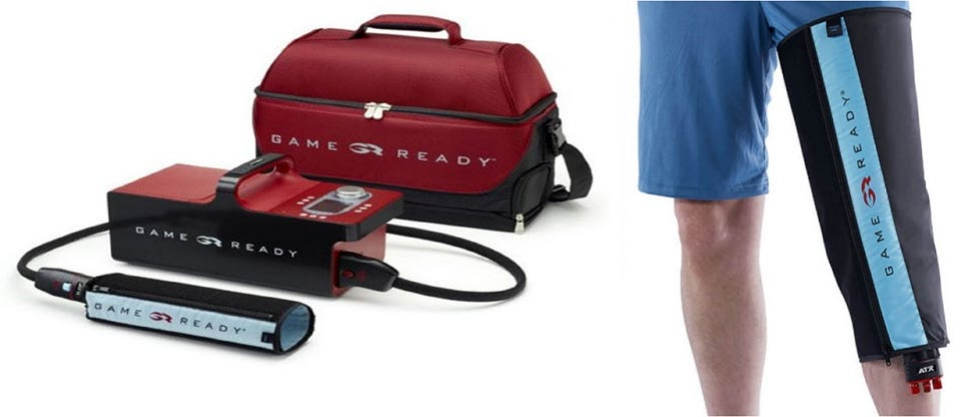
GRPro^®^ 2.1 system and straight knee wrap used by the GR group

Both treatment groups commenced their intervention on day 0 upon their return to the ward after their TKA and continued for a period of two weeks (spanning the in-patient and early out-patient setting). The UC group underwent regular icing (bag of crushed ice) along with tubigrip static compression (on average 17 mmHg) [24] applied under the ice, as per the routine hospital and early out-patient pathway. The GR device, which received approval from the Australian Government Department of Health Therapeutic Goods Administration (TGA) for clinical use in 2008, delivers cryotherapy with intermittent compression via a circumferential wrap (Fig. 1), encompassing the leg from the thigh to the calf. Patients randomised to the GR group were provided their own leg wrap which they used for the duration of their intervention (2 weeks). Prior research has found there to be 2.5°–3.5° difference in intramuscular temperatures between the two modalities, with crushed ice providing more cooling [2, 12]. In these studies, the ice was directly applied onto the skin, hence by applying the tubigrip under the ice we aim to eliminate this temperature difference.

Both groups followed a 2-week protocol of either ice plus tubigrip (UC) or use of the GR device for a period of 20 min, 6 times a day, with an off time of at least one hour between applications. Specific application times were not set but the regime was to commence first thing in the morning, and with patients requiring a one hour “off” period between sittings the intervention times were dispersed between morning, afternoon and evening. Patients were provided a logbook to keep track of their routine and to assist them in complying to the frequency protocol. The GR device was set on a low pressure (cycle of 5–15 mmHg) for days 0–5 and then a medium pressure (cycle of 5–50 mmHg) from days 6–14. A standardised temperature of 5 °C was used which was well tolerated by all patients. The physiotherapist initially set up the GR device controls for the patient in hospital and instructed them how to use and adjust the system as needed. While in hospital both interventions were initiated by nursing and physiotherapy staff and then carried on by the patients themselves on discharge.

### Baseline measurements

Pre-operative baseline demographic characteristics of age and gender were recorded, along with active knee ROM (flexion and extension) and knee circumference at the mid-patella point, as detailed further below. Pre-operative ROM measurements were taken with a hand-held goniometer by the treating orthopaedic surgeon which has shown to have high inter-rater and intra-rater reliability [11]. One week prior to their TKA a baseline Knee Injury and Osteoarthritis Outcome Score (KOOS) questionnaire [8] was completed and scored from 0 (worst) to 100 (best), as was a baseline Visual Analogue Pain Scale (VAS).

### Post-operative clinical outcomes

A range of subjective and objective measures were collected throughout the post-operative period. For early post-operative subjective measures, patients were provided with a study logbook to record pain (VAS) scores and medication usage over the first 2 weeks, which was subsequently collected from patients at their 2-week out-patient appointment. The VAS was used to assess patients’ pain levels, on a whole number rating scale from 0 (no pain) to 10 (worst pain). VAS was recorded at day 1, day 2, week 1 and week 2, and subsequently at the patient’s 6-week out-patient appointment. Opioid usage was recorded from in-patient medication charts and then from the patient’s logbook following hospital discharge, converted to milligram of morphine equivalents for standardisation. In-patient hospital LOS was recorded upon discharge.

KOOS was assessed at 6-weeks post-surgery. Given the early timeframe that the study was focused on, only the overall KOOS score has been reported, along with subscales for Pain, Symptoms and QOL. A Patient Satisfaction Questionnaire (PSQ) was also employed to evaluate the patient’s level of satisfaction with their method of cryocompression therapy and its ability to relieve their pain and improve their ability to perform daily activities. It also assessed their satisfaction in ease of use and application, perceived swelling control, sleep and mobility. A categorical tool was devised: 1 = very satisfied; 2 = somewhat satisfied; 3 = somewhat dissatisfied; 4 = very dissatisfied, with an overall score between 9 (best) to 36 (worst). These were completed at the 2-week, 6-week and 3-month post-operative time-points.

Objective measurements collected in hospital were non-blinded and undertaken and recorded by the patient’s treating physiotherapist, commencing on day 1 after the bulky crepe compression bandage was removed. Ongoing knee ROM and circumference measurements were taken and recorded by a blinded research assistant at follow-up appointments in the orthopaedic surgeon’s private practice. Both active knee flexion and extension ROM were recorded using a hand-held long-arm goniometer which has shown high test–retest reliability following knee arthroplasty with an intraclass correlation (ICC) of 0.99 [31]. Knee circumference measurements were taken to assess knee swelling, assessed at the mid-patella point. These were all undertaken at day 1, day 2, week 2 and week 6 post-operatively.

Ethics approval was provided by the Hollywood Private Hospital Research Ethics Committee, approval number HPH542.

### Statistical analysis

An a priori power calculation was undertaken for the primary outcome measure (VAS difference of 1 point at 2-weeks post-surgery), determined based on the recommendations of Cohen [7]. Based on preliminary data collected as part of a pilot study, this indicated that for an anticipated large effect size (*d* = 0.9), a total of 52 patients (27 in each group) would be required to reveal differences at the 5% significance level, with 90% power. Data were summarised using mean and standard deviation (SD). The PROMs, hospital LOS and mid-patella circumference were compared between the groups (UC and GR) using two tailed t-tests. The remainder of the outcomes were treated as continuous and modelled with a linear mixed model with random intercepts for patients. Using linear modelling analysis enabled interpretation and analysis of variables where there were higher amounts of missing follow-up data. Where possible, a random effect for surgeon was fitted to allow for inter-surgeon variability. Fixed effect terms were fitted for the time by treatment interaction to assess whether the outcome varied over time differently in each group. Overall significance of each outcome was calculated over time (including pre-operative measurements) using Analysis of Variance (ANOVA) with Satterthwaite’s method from the linear mixed models. The overall test for an interaction is reported along with marginal contrasts comparing the two groups where appropriate. Patient age and gender were assessed as potential confounders; in the case of the post-operative opioid outcome, pain was also assessed as a confounder. Statistical analysis was performed using R [25] with lme4 [4] and statistical significance was set at *p* < 0.05.

## Results

**Fig. 2:**
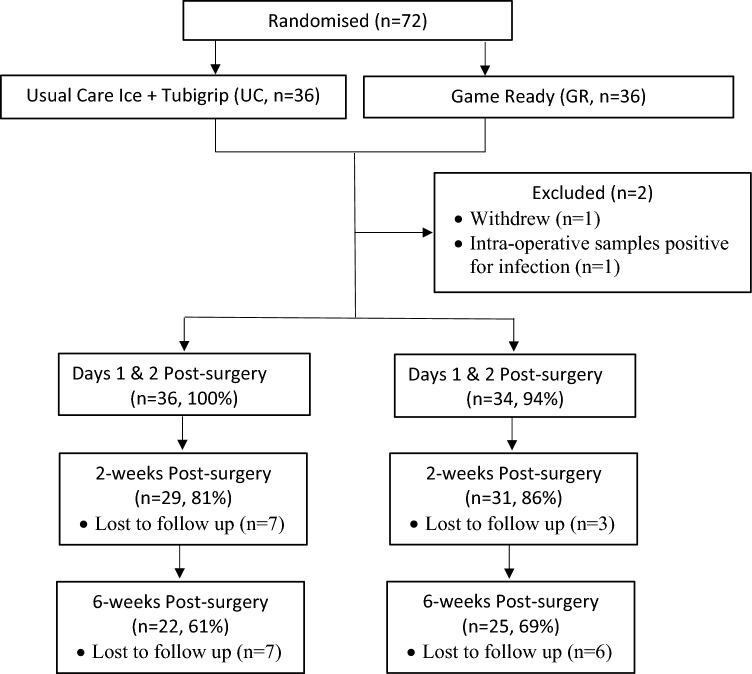
Flowchart of patient recruitment, randomisation and dropout rates follow-up

A total of 72 knees (36 per group) in 70 patients were recruited for the study between January 2020 and November 2021 (Fig. 2). The two patients that had both knees in the study were not undertaken as bilateral TKAs, rather these patients underwent subsequent TKA (and further randomisation) on the contralateral knee within the study recruitment period. One patient withdrew immediatley post-surgery while one further patient was excluded due to intra-operative tissue samples being positive for infection, thus rendering a different post-operative pathway for this patient. The GR group had 7 patients (19%) lost to follow-up at 2 weeks and another 7 (19%) by the 6 week post-operative mark. The UC group had 3 patients (8%) lost to follow-up at 2 weeks and another 6 (16%) by the 6 week post-operative mark. Compliance to treatment protocol was high, with all patients stating they followed the instructed regimen and 92% and 95% of the UC and GR group, respecitvely, returning their completed log booklets. There were no pre-operative differences between the two groups (n.s.) in demographics, VAS, knee ROM or circumference measures (Table 1).

**Table 1:** Pre-operative demographics, visual analogue pain scale (VAS), knee range of motion (ROM) and circumference measures between the game ready (GR) and usual care (UC) groups

Variable	UC (*n* = 36)	GR (*n* = 36)	*p* value
Gender: *n* (%)			
Female	17 (47.2%)	23 (63.9%)	n.s.
Male	19 (52.8%)	13 (36.1%)	
Age	68.9 (8.79)	64.7 (10.1)	n.s.
VAS	4.84 (1.81)	5.82 (2.56)	n.s.
ROM (degrees)			
Flexion	130 (8.37)	126 (11.6)	n.s.
Extension	4.88 (3.6)	7.19 (7.15)	n.s.
Knee circumference (cm)	41.7 (3.57)	42.2 (3.77)	n.s.

Shown are means (SD)

There were no significant group-by-time interactions regarding any of the outcomes. No significant differences (n.s.) were found between the two groups in the VAS at any post-operative time-point (Table 2). Males in both groups (compared with females) reported significantly less (*p* = 0.048) pain on the VAS. No significant difference (n.s.) at days 1–3 post-operatively or overall in opioid usage was observed between the two groups (Table 2). After adjusting for age and gender, increased pain was associated with increased opioid use (*p* < 0.001) across all patients. Furthermore, older age was significantly associated (*p* < 0.001) with lower opioid usage, even after adjusting for pain use.

**Table 2:** Predicted values from linear mixed model Analysis at post-operative time-points for the visual analogue pain scale (VAS), opioid consumption and knee range of motion (ROM), for both the usual care (UC) and game ready (GR) groups

Variable	UC	GR	*p* value
VAS			
Day 1	5.0 [1.2, 8.8]	5.5 [1.7, 9.3]	n.s.
Day 2	4.0 [0.3, 7.8]	4.7 [0.9, 8.4]	n.s.
Week 1	4.2 [0.3, 8.0]	4.3 [0.5, 8.1]	n.s.
Week 2	2.6 [1.2, 6.4]	2.8 [− 1.0, 6.6]	n.s.
Week 6	2.1 [− 1.9, 6.1]	2.6 [− 1.2, 6.5]	n.s.
Opioid consumption (mg of morphine equivalents)			
Day 1	70.1 [4.5, 135.7]	82.3 [17.0, 147.6]	n.s.
Day 2	73.9 [8.3, 139.5]	83.3 [18.0, 148.7]	n.s.
Day 3	68.5 [3.0, 134.1]	74.1 [8.8, 139.5]	n.s.
Extension ROM (degrees)			
Day 1	6.1 [− 1.5, 13.7]	5.6 [− 2.0, 13.2]	0.048
Day 2	4.7 [− 2.9, 12.3]	4.8 [− 2.8, 12.4]	n.s.
Week 2	5.1 [− 2.5, 12.8]	3.5 [− 4.1, 11.2]	0.007
Week 6	3.1 [− 4.8, 10.9]	3.3 [− 4.4, 11.0]	n.s.
Flexion ROM (degrees)			
Day 1	65.1 [42.8, 87.4]	70.1 [47.8, 92.3]	n.s.
Day 2	70.0 [47.7, 92.2]	68.7 [46.9, 90.9]	n.s.
Week 2	86.6 [64.2, 109.0]	87.1 [64.9, 109.3]	n.s.
Week 6	103.0 [80.2, 125.9]	94.8 [72.3, 117.4]	n.s.

Results displayed as mean [confidence intervals]

The GR group had significantly 0.5° more knee extension ROM than the UC group at day 1 (*p* < 0.05) and 1.6° day 14 (*p* < 0.01) post-surgery (Table 2). No differences (n.s.) were observed between groups in active knee flexion ROM (Table 2) or knee circumference (Table 3) measures. While the GR group reported significantly worse (*p* < 0.05) KOOS scores pre-operatively, these were not statistically worse (n.s.) post-surgery, with the GR group demonstrating a larger pre- to post-surgery percentage improvement in each KOOS domain. There were no statistically significant (n.s.) group differences in the PSQ between groups (Table 3). There were no adverse events observed relating to either group’s intervention.

**Table 3:** Length of hospital stay, mid-patella circumference, the patient satisfaction questionnaire (PSQ) and the knee injury and osteoarthritis outcome score (KOOS), for both the usual care (UC) and game ready (GR) groups

Variable	UC	GR	Difference	*p* value
Length of hospital stay (nights)	4.7 (1.6)	4.8 (1.1)	0.1	n.s.
Mid-patella circumference (cm)				
Day 1	45.2 (5.6)	46.9 (5.3)	1.7	n.s.
Day 2	46.2 (4.4)	46.3 (5.8)	0.1	n.s.
Week 2	45.5 (3.8)	46.6 (7.4)	1.1	n.s.
Week 6	45.0 (4.1)	43.8 (4.9)	1.2	n.s.
PSQ (9 = best, 36 = worst)				
Week 2	14 (5.1)	12.5 (2.1)	1.5	n.s.
Week 6	15.2 (3)	11.2 (1.3)	4	n.s.
KOOS—overall (%) (100 = best)				
Pre-operatively^a^	54.5 [31–79]	46.6 [27–76]	7.9	0.024
6 weeks^a^	75.9 [65–95]	69.6 [39–97]	6.3	n.s.
Individual % improvement in score	40 (13.8)	52 (20.1)	12	n.s.
KOOS—pain (%) (100 = no pain)				
Pre-operatively^a^	57.9 [39–83]	50.4 [28–83]	7.5	0.035
6 weeks^a^	82.8 [70–100]	73.4 [42–100]	9.4	n.s.
Individual % improvement in score	47 (13.6)	53 (23.9)	6	n.s.
KOOS – Symptoms (%) (100 = none)				
Pre-operatively^a^	53.7 [25–86]	44 [18–68]	9.7	0.017
6 weeks^a^	76.4 [50–100]	72.1 [54–99]	4.3	n.s.
Individual % improvement in score	43 (28.9)	56 (21.1)	13	n.s.
KOOS—quality of life (%) (100 = best)				
Pre-operatively^a^	45.5 [19–87]	33.5 [0–62]	12	0.009
6 weeks^a^	57.5 [25–69]	63 [25–98]	5.5	n.s.
Individual % improvement in score	15 (19.4)	45 (22.5)	30	n.s.

Shown are means (SD)

^a^Results shown as mean [range]

## Discussion

The most important outcome from the current study was that patients using the GR cryocompression device over the first two post-operative weeks after TKA demonstrated better knee extension ROM compared with a UC ice and tubigrip protocol. Despite reaching statistical significance, due to the high dropout rate, this finding may be a result of chance. Our findings correlate to those of Ueyama et al. [30] where a cooler knee following TKA lead to improved ROM. Regaining knee extension following TKA is important in the restoration of gait and has been shown to correlate with superior PROMs [37], with every 1 degree of extension gained leading to an improved in Oxford knee score by a factor of 0.11 [37]. There is still no consensus as to what extra degree of ROM is beneficial [35] but a study by Zeni et al. found that there is a 23% increased likelihood of undergoing TKA for every one degree less of extension ROM [34]. The success of TKA is often measured based on the restoration of knee ROM [20].

Previous literature evaluating the GR system have used differing treatment protocols and this may have contributed to the variation in our findings. No previous studies have found any significant improvements in knee ROM. Su et al. [23] applied the GR for a period of two hours while in hospital and one hour following discharge, four times a day for a two week period and found a significant reduction in the use of opioid analgesia and a higher level of patient satisfaction. In comparison, Murgier et al. [21] applied the GR for two 8-h cycles per day for the first three post-operative days and found a significantly lower pain score on the third day. Both studies applied the GR for much longer periods but fewer times per day than the treatment regime in the current study. Patient factors such as the amount of adipose tissue can also affect tissue cooling with 25 min being adequate to cool an area with a skinfold of 20 mm versus 60 min for a skinfold of 30-40 mm [22]. This may indicate that we have not yet found the optimal protocol to be used with cryocompression therapy to gain the best results and this is an area which would benefit from further investigation. The finding of improved ROM occurred while the intervention was being implemented. There may be rationale to suggest the use of GR for longer, up to 6 weeks in the acute post-operative period, with Komatsu et al. showing post-operative effects lasting over 6 weeks in rats who underwent TKA, with weight bearing alterations lasting up to 35 days [13].

The lack of significant differences between the two groups rejected the study hypothesis. There is an associated cost with use of GR compared to regular icing which must be considered, so although our results show marginal favour towards the use of GR it would be more suited to patients in the private hospital setting. Through the mixed model statistical analysis using gender and age as confounding factors, we discovered that males significantly reported on average less pain on the VAS than females and older patients consumed less opioids. These findings align with previous literature demonstrating a higher prevalence and experience of pain in females [3]. When analysing the pre-operative demographics between the two groups, the UC group had 21% more males, were on average 4.2 years older and had a baseline VAS which was 0.98 points lower than the GR group. This may have skewed the group findings, though were also the result of randomisation.

It can be difficult to ascertain if a patient is truly happy and satisfied with the outcome of their TKA. There is a large amount of scope for interpretation of questions posed in PROMs which can lead to variation in results and ultimately may not be the best measurement for patient satisfaction [6]. We endeavoured to combat this by formulating our own PSQ to evaluate patients’ perception in satisfaction of their given method of cryocompression therapy. At the 6-week post-operative mark, the GR group on average scored 4 points higher regarding their satisfaction, though the lack of statistical significance may indicate that the study was underpowered for this domain. Patient satisfaction is of the upmost importance and ultimately determines the patient’s perception of whether their intervention was a success, which may not always correlate with clinical outcome measures [26].

Several study limitations are acknowledged. Firstly, study onset and recruitment coincided with the onset of the COVID-19 pandemic which impacted data collection due to reduced patient clinic attendance. Losses to follow-up over the 6-week post-operative period could imply that our significant results are due to chance and thus cannot be interpreted as being clinically significant. These circumstances are not unique to our study with the COVID-19 effect on clinical trials being well documented [32]. Secondly, it was not possible to blind participants given the nature of the study and protocols employed. Subjective reporting was not statistically different between groups, however. Thirdly, while all pre-operative and out-patient post-operative assessments were undertaken by the same researcher, the timing of in-patient assessments (i.e., morning or afternoon) could not be standardised and assessment by the same inpatient physiotherapist was not possible. Fourthly, the current study employed an initial post-operative 2-week intervention period only, and future research may investigate the additional benefit of a longer period employing the GR protocol. Finally, we acknowledge the short-term assessment period (6 weeks) of the study and, while longer-term review may be warranted, the study was designed to assess the role of the GR device on the early acute period after TKA.

## Conclusion

Use of a cryocompression device following total knee arthroplasty is a safe, non-invasive tool that may aid in the post-operative recovery period. Despite patients gaining significantly more knee extension during the initial two-week intervention period when using GR compared to UC, this effect was likely due to chance. No further significant differences were observed between the groups during or after cession of the intervention.
